# Secretion of Biologically Active Heterologous Oxalate Decarboxylase (OxdC) in *Lactobacillus plantarum* WCFS1 Using Homologous Signal Peptides

**DOI:** 10.1155/2013/280432

**Published:** 2013-07-18

**Authors:** Ponnusamy Sasikumar, Sivasamy Gomathi, Kolandaswamy Anbazhagan, Govindan Sadasivam Selvam

**Affiliations:** ^1^Department of Biochemistry, Centre for Advanced Studies in Organismal and Functional Genomics, School of Biological Sciences, Madurai Kamaraj University, Madurai 625 021, India; ^2^INSERM-U844, Institut des Neuroscience de Montpellier Building, Hopital St. Eloi, 34091 Montpellier, France

## Abstract

Current treatment options for patients with hyperoxaluria and calcium oxalate stone diseases are limited and do not always lead to sufficient reduction in urinary oxalate excretion. Oxalate degrading bacteria have been suggested for degrading intestinal oxalate for the prevention of calcium oxalate stone. Here, we reported a recombinant *Lactobacillus plantarum* WCFS1 (*L. plantarum*) secreting heterologous oxalate decarboxylase (OxdC) that may provide possible therapeutic approach by degrading intestinal oxalate. The results showed secretion and functional expression of OxdC protein in *L. plantarum* driven by signal peptides Lp_0373 and Lp_3050. Supernatant of the recombinant strain containing pLp_0373sOxdC and pLp_3050sOxdC showed OxdC activity of 0.05 U/mg and 0.02 U/mg protein, while the purified OxdC from the supernatant showed specific activity of 18.3 U/mg and 17.5 U/mg protein, respectively. The concentration of OxdC protein in the supernatant was 8–12 **μ**g/mL. The recombinant strain showed up to 50% oxalate reduction in medium containing 10 mM oxalate. In conclusion, the recombinant *L. plantarum* harboring pLp_0373sOxdC and pLp_3050sOxdC can express and secrete functional OxdC and degrade oxalate up to 50% and 30%, respectively.

## 1. Introduction 

Oxalate is found in a variety of foodstuffs including pepper, parsley, spinach, rhubarb, cocoa, tea, certain dark green leafy vegetables, and plants and those who consume excess dietary oxalate rich food may develop nephrolithiasis. Under normal conditions, the daily load of oxalate, derived from endogenous production and intestinal absorption is fully excreted by the kidneys. Elevated oxalate level in urine known as hyperoxaluria is one of the major risk factors for recurrent urolithiasis and progressive nephrocalcinosis [1]. In addition, several pathological conditions, including Crohn's disease, steatorrhea, and cystic fibrosis, or medical procedures such as jejuno-ileum bypass surgery are associated with enteric hyperoxaluria due to enhanced oxalate absorption in the colon [2–4]. The lack of new medications and the continued poor compliance with drug therapy have led to a growing interest in dietary manipulation and novel therapies aimed at preventing recurrent stone formation. Unfortunately, an oxalate-free diet is difficult to achieve and would probably be deficient in essential nutrients. Hence, other approaches to reducing urinary oxalate for management of stone disease have been explored. Normally, the intestinal tract is colonized with bacteria such as *Oxalobacter formigenes* (*O. formigenes*), *Lactobacillus* species, and others [5, 6]. Intestinal oxalate degrading bacteria are capable of degrading oxalate to CO_2_ and formate; the latter is further metabolized and excreted via feces. Thus, treatment with oxalate degrading bacteria could be a new therapeutic option in patients with hyperoxaluria and calcium oxalate stone disease. Although oxalotropic bacterium, *O. formigenes *is efficient in oxalate degradation [7–9], its probiotic efficiency is still uncertain and not as well established as in lactic acid bacteria (LAB). In addition, colonization of *O. formigenes *may require oral oxalate supplements to the gut since it is a strict oxalotrophic bacterium.

Probiotics have been evaluated in animals and humans with respect to antibiotic-associated diarrhea, traveler's diarrhea, pediatric diarrhea, inflammatory bowel syndrome, and irritable bowel syndrome [10]. Over the past decade there has been increasing interest in the use of LAB as a delivery system for a range of different applications, including anti-infective, allergic diseases, and gastrointestinal diseases [11]. Del Rio et al. [12] developed an oral vaccine based on *Lactobacillus plantarum* expressing the outer surface protein A (OspA), a lipoprotein from *Borrelia burgdorferi*. Evidence suggests that LAB play a role in controlling intestinal microbiota, restoring intestinal barrier function and alleviating inflammatory responses. It is also used in the therapy aid management of immunological disorders such as Crohn's disease and Pouchitis [13, 14]. Genetically modified LAB such as *Lactococcus lactis* have been used for delivery of interleukin-2, -6, and -10 to combat murine colitis [15, 16]. *Lactobacillus casei* has been used for the delivery of *β*-lactoglobulin in the digestive tract of mice [17]. Similarly, modified *Lactobacillus* was shown to protect against dental caries in rat [18].

Earlier reports showed that wild type LAB have no influence on reduction of urinary oxalate excretion [19]. The use of oxalate decarboxylase (OxdC) enzyme to decompose intestinal oxalate was broadly demonstrated. Orally given recombinant *Bacillus subtilis* (*B. subtilis*) OxdC expressed in *Escherichia coli* (*E. coli*) has substantially declined the urinary oxalate level in an experimental rat model [20]. Oral therapy with crystalline, cross-linked formulation of the OxdC, was demonstrated to diminish symptoms of hyperoxaluria and able to prevent nephrocalcinosis and urolithiasis in mice [21]. Further, orally given formulation of *B. subtilis* OxdC, was shown to be safe in rats and dogs during short-term toxicity tests [22]. Thus, it can be hypothesized that manipulation of lactic acid bacterial component of intestinal microflora with heterologous expression of oxalate degrading protein (OxdC) may decrease the intestinal oxalate and correspondingly intestinal oxalate absorption and renal excretion.

Recently, in our laboratory the LAB that heterologously express OxdC were developed for the possibility of utilizing as a potential probiotics for depletion of the intestinal dietary oxalate [23]. Since the expression of OxdC was at the intracellular level the degradation of external oxalate was limited. Therefore, further modification with a desired promoter and secretary elements for its extracellular expression of protein (OxdC) towards the alleviation of hyperoxaluria is vital. The functionality of homologous signal peptides (SPs) sequences of *Lactobacillus plantarum* WCFS1 (*L. plantarum, *isolate from human saliva) was studied by Mathiesen et al. [24]. The study showed that predicted signal peptides in the *L. plantarum* genome actually are capable of driving the protein secretion. In the present work, we investigated the secreting efficiency of OxdC protein in human microbiota strain *L. plantarum* by using sakacin P controlled *Lactobacillus* derivatives of the expression vector pSIP401, combined with the homologous SPs sequence Lp_0373 and Lp_3050 to drive the secretion of OxdC. The extracellular level expression, biological activity of recombinant OxdC, and oxalate degrading efficiency of the recombinant *L. plantarum* were evaluated in this study.

## 2. Materials and Methods

### 2.1. Bacterial Strains, Media, and Growth Conditions

The bacterial strains and plasmids used in this study are listed in Table 1. *E. coli* DH10B (Invitrogen) was grown in Luria-Bertani (LB) broth at 37°C with shaking and *L. plantarum* WCFS1 was grown in deMan-Rogosa-Sharpe (MRS) media at 30°C without shaking. Solid media were prepared by adding 1.5% (w/v) agar to the broth. Erythromycin (Em) was added at a final concentration of 200 **µ**g/mL for *E. coli* and 5 **µ**g/mL for *L. plantarum*.

### 2.2. Standard DNA Techniques and Transformation

All cloning and transformation steps were carried out using standard methods [27]. Peptide and primers used in this study were synthesized and procured from Sigma Aldrich, USA (Table 2). Taq DNA polymerase and the PCR product purification kit was purchased from Sigma-Aldrich, USA. Restriction enzymes *Sal *I, *Eco*R I, and T4 DNA Ligase were obtained from Fermentas (Thermo Scientific, USA). Restriction enzyme digested DNA was purified with Gen elutes gel extract kit (Sigma Aldrich, USA). The PCR amplified products were verified by DNA sequencing (Eurofins Genomics India Pvt., Ltd, India). *E. coli *DH10B was used as host strain for maintaining plasmids. *L. plantarum* was electrotransformed as described elsewhere [28].

### 2.3. Manipulation of Recombinant Plasmids

Expression vectors constructed in this study are based on pSIP401, a 5.67-Kb vector developed for inducible gene expression and expression of the gene of interest regulated by a two-component regulatory system (SppK & SppR), which is activated by an externally added peptide pheromone [29, 30]. In pSIP401 vector, the homologous signal peptide sequence Lp_0373 and Lp_3050 of *L. plantarum* WCFS1 was cloned at downstream of the sakacin P promoter (P_*sppA*_), and its functionality was studied and discussed elsewhere [24, 31]. In the present work the *oxdC* gene was amplified from plasmid pLB36 [26] by using OXDC sense and OXDC antisense primer harboring the *Sal *I and *Eco*R I sites, respectively. The PCR program consisted of an initial denaturation at 95°C for 5 min and 40 cycles of 95°C for 30 s and 58°C for 1 min and extension for 72°C for 1 min followed by final extension at 72°C for 10 min. The 1.2 kb PCR product was digested with *Sal *I and *Eco*R I and ligated into *Sal *I/*Eco*R I digested pLp_0373sAmyA and pLp_3050sAmyA, yielded pLp_0373sOxdC and pLp_3050sOxdC, respectively. The final recombinant vector size of 6.9 Kb for pLp_0373sOxdC and pLp_3050sOxdC was confirmed by colony PCR, restriction analysis, and sequencing using primer PsppAF. These expression vectors were constructed in *E. coli* DH10B before electroporation into the expression host *L. plantarum *WCFS1. Our laboratory vector construct pSip-OxdC that produces intracellular OxdC was used as control [23].

### 2.4. SDS-PAGE Overexpression Analysis of OxdC in Recombinant *L. plantarum *WCFS1

Protein profiles in the cell-free supernatants and purified proteins were visualized by running 12% separating gel and 4% stacking gel of sodium dodecyl sulfate polyacrylamide gel electrophoresis (SDS-PAGE) [32]. The heterologous OxdC proteins in recombinant *L. plantarum* WCFS1 strains were induced by sakacin P (SppIP) as described by Mathiesen et al. [33]. Briefly, the overnight cultures of the clones containing plasmids pLp_0373sOxdC, pLp_3050sOxdC and pSip-OxdC were subcultured in MRS medium containing 5 mM MnCl_2_. The cultures were allowed to grow till the absorbance reaches OD_600_ ~ 0.3 and they were induced by adding SppIP to a final concentration of 25–50 ng/ml. Cell pellet and the supernatant were collected separately at OD_600_ of ~1.7. The protein present in cell free supernatant was precipitated by adding ammonium sulphate at a final concentration of 75%. Further, the precipitated protein was pelleted by centrifugation at 16000 rpm for 30 min at 4°C. The pellet was resuspended in 1/15th volume of TES buffer (10 mM Tris-HCl + 1 mM EDTA + 25% sucrose) and analyzed on an SDS-PAGE along with protein marker. The proteins were visualized by silver staining and the gel was stored in 1% glacial acetic acid.

### 2.5. Assay for OxdC Activity from Culture Supernatant Expressed from pLp_0373sOxdC and pLp_3050sOxdC

Oxalate decarboxylase activity was assayed in both cell free supernatants and cell lysate of wild type and recombinants *L. plantarum* WCFS1 strains independently. The heterologous OxdC proteins in recombinant *L. plantarum* strains were induced as described elsewhere in this paper. When the OD_600_ reaches ~1.7, the cells were harvested and the protein in culture supernatant was concentrated by adding 75% ammonium sulphate. Further, the precipitated protein samples were dialyzed against 50 mm Tris-HCl (pH 8.5) and 0.5 M NaCl at 25°C for overnight. The OxdC assay was performed as described by Tanner and Bornemann [34]. In brief, an appropriate volume of reaction mixture containing 10 mM sodium citrate (pH 4.0), 150 mM potassium oxalate, was taken in 1 mL cuvette and concentrated supernatant was added to the mixture at 25°C and incubated for 2 min. The mixture was neutralized with phosphate buffer (pH 9.5). Subsequently, NAD was added and was monitored for its reduction in a spectrophotometer (Hitachi) at 340 nm in the presence of formate dehydrogenase (Sigma Aldrich). One unit of enzyme activity was defined as the amount of enzyme required to reduce 1 **µ**M of NAD per min. Total protein concentration was measured by biuret method using protein assay kit (Reckon Diagnostics Pvt., Ltd, India) in semiautomated photometer 5010 V5+ (Robert Riele GmbH, Germany) against the bovine serum albumin as a standard. Similarly, the OxdC activity was also measured for corresponding strains at early exponential growth (OD_600_ ~ 0.5) and late exponential growth (OD_600_ ~ 1).

 To measure the intracellular OxdC activity, cell pellets were washed once with double distilled water and suspended in sodium citrate buffer (pH 4.0) and disrupted by sonication. The cell free extract was obtained by centrifugation at 16000 rpm for 30 min at 4°C. The assay for OxdC was performed. To assess the secretion efficiency, intracellular OxdC activity was measured and compared with extracellular activity as described elsewhere [31]. OxdC activity for the control strain was also assayed.

### 2.6. Purification of OxdC from Culture Supernatant of Recombinant *L. plantarum* WCFS1

Supernatant containing recombinant His tag-fused OxdC protein was purified by FPLC system (BioLogic DuoFlow, Bio-Rad) equipped with 5 mL profinity column and enzymatic assay was carried out. Briefly, the ammonium sulphate precipitated proteins were collected by centrifugation at 16000 rpm for 30 min at 4°C. The protein pellet was dissolved in buffer containing 50 mM imidazole-HCl (pH 7.0), 1 M sodium chloride, 10 **μ**M MnCl_2_, and 1 mM of protease inhibitor Phenylmethyl Sulfonyl fluoride (PMSF). It was further loaded into previously washed 5 mL profinity column. The column was equilibrated with 50 mM phosphate buffers (pH 8.0) containing 0.5 M NaCl_2_ and 20 mM imidazole. OxdC was eluted using 0.02–0.5 M imidazole gradient. The flow rate was maintained at 1 ml/min. Fractions of purified proteins were collected in different collection tube and analyzed on 12% SDS-PAGE along with the protein marker and protein was visualized by Coomassie brilliant blue staining. The fractions containing purified OxdC were pooled and dialyzed against 50 mM Tris-HCl, pH 8.5 containing 0.5 M NaCl. The purified enzyme was stored at −80°C for later use. The protein quantification and assay for OxdC were performed as described elsewhere in this paper. Specific activity was calculated as the mean value of three independent assays.

### 2.7. *In Vitro* Analysis of Oxalate Degradation by the Recombinant *L. plantarum* Expressing Heterologous OxdC

The oxalate degrading ability of recombinant *L. plantarum* WCFS1 and respective controls were examined at various time intervals. In brief, bacterial culture was grown in MRS medium supplemented with 10 mM potassium oxalate up to OD_600_ ~ 0.3. Expression of OxdC was induced by adding 50 ng of induction peptide (SppIP) and 5 mM of MnCl_2_. Cell-free medium was collected at different intervals and assayed for oxalate concentration using oxalate oxidase enzymatic methods (Trinity Biotech, Ireland) according to the manufacturer's instructions in semiautomated analyzer photometer 5010 V5+ (Robert Riele GmbH, Germany) at OD 590 nm. Experiments were repeated three times and values were expressed as mean.

## 3. Results

### 3.1. Construction of Secretion Vectors for the Heterologous Expression of OxdC in *L. plantarum* WCFS1

**Figure 1:**
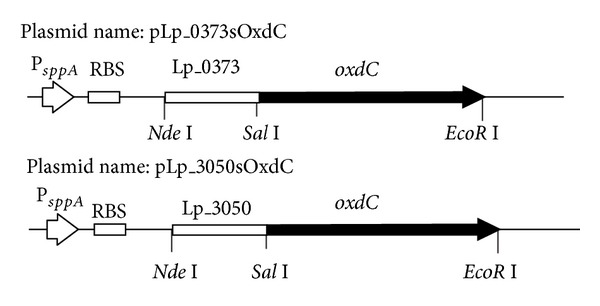
Schematic representation of expression cassette for production and secretion of oxalate decarboxylase under the control of promoter (P_*sppA*_) in *L. plantarum* for details of plasmid constructions; see the text and Table 1. P_*sppA*_ indicates the sakacin-P inducible promoter, RBS indicates ribosomal binding site, Lp_0373 and Lp_3050 indicate the signal peptides sequence, *oxdC* indicated the oxalate decarboxylase gene, and restriction sites between the signal peptide sequences and *oxdC *gene also are indicated.

For the construction of OxdC secretion vector in *L. plantarum *WCFS1, we selected two different plasmid pLp_0373AmyA and pLp_3050AmyA, which harbor homologous signal peptide sequence (Lp_0373 and Lp_3050) of *L. plantarum* at downstream of the sakacin P promoter (P_*sppA*_). In addition, both the signal peptides functionality were well studied in homologous host *L. plantarum *WCFS1 [24, 31]. Their efficiency of secretion and higher activity of secreted protein prompted us to select these two signal peptides (Lp_0373 and Lp_3050) to study the secretion analysis of OxdC in *L. plantarum*. The resulting recombinant vectors have the size of 6.9 Kb (pLp_0373sOxdC and pLp_3050sOxdC). Standard modular expression cassettes developed are depicted in Figure 1. The vectors were first established in *E. coli* before electroporation into *L. plantarum*.

### 3.2. Heterologous Production and Functional Expression of OxdC by Recombinant *L. plantarum* WCFS1

**Figure 2:**
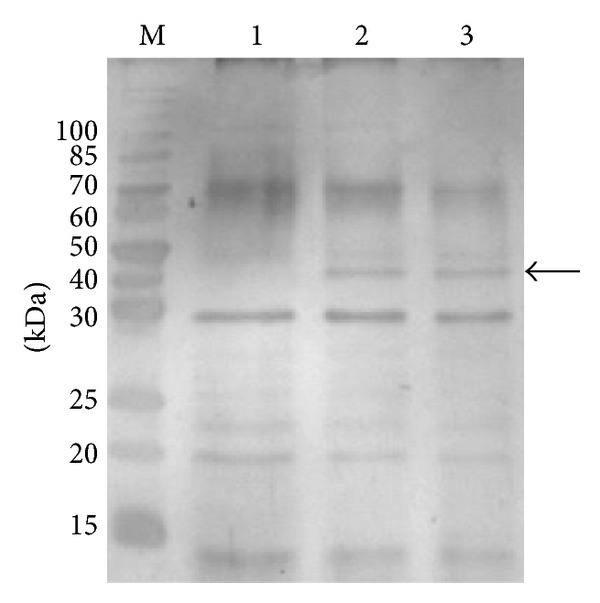
Silver stained gels after SDS-PAGE of supernatant of *lactobacillus* strain harboring various plasmids. All cells were induced with 50 ng inducing peptide mL^−1^ at OD_600_ ~ 0.3 and harvested at OD_600_ ~ 1.7; lane M, molecular weight marker (kDa); lane 1, strain harboring pSIP-OxdC; lane 2, pLp_3050sOxdC; lane 3 pLp_0373sOxdC; arrow indicate, the location of OxdC protein (calculated molecular mass of 44 kDa).

The samples of supernatants were taken in late logarithmic phase (OD_600_ ~ 1.7) of recombinant *L. plantarum* WCFS1 containing the vectors pLp_0373sOxdC, pLp_3050sOxdC, and pSip-OxdC. SDS-PAGE analysis of concentrated supernatants showed protein bands equivalent to the 44 kDa of OxdC by silver staining for the strain harboring secretion plasmids pLp_0373sOxdC and pLp_3050sOxdC. However, the 44 kDa could not be detected on recombinant strain carrying plasmid pSip-OxdC (Figure 2). This shows the secretion of OxdC in supernatant, by signal sequences Lp_0373 and Lp_3050, but not in the control strain containing the plasmid pSip-OxdC due to the lack of signal peptide sequences.

**Figure 3:**
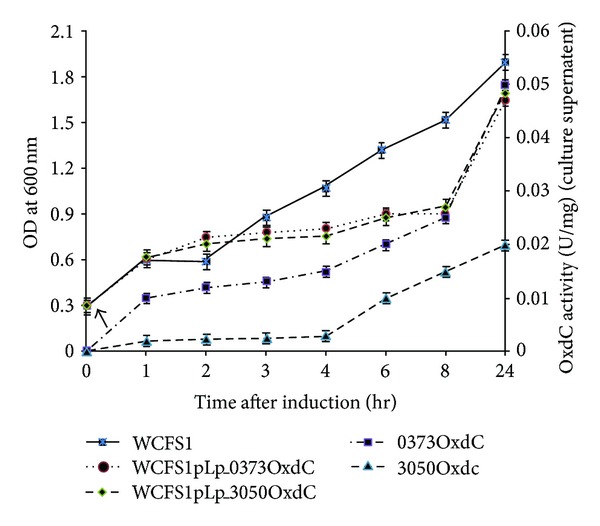
Growth curve analysis and OxdC activity in culture supernatant of different recombinants *L. plantarum* construct against nonrecombinant *L. plantarum* after induction (arrow indicates the induction point at OD_600_ ~ 0.3). The represented data are mean value of three independent experiments; 0373OxdC (solid square) and 3050OxdC (solid triangles) indicates the enzymatic activity of OxdC in culture supernatant during corresponding growth time OD_600_ ~ 0.5 (1st hr) late exponential growth OD_600_ ~ 1 (6th hr) and late logarithmic phase OD_600_ ~ 1.7 (24th hr) of recombinant strains WCFS1pLp_0373OxdC, WCFS1pLp_3050OxdC, respectively, and WCFS1 indicates the wild type strain of *L. plantarum*.

OxdC activity in the concentrated cell-free supernatant of recombinant strains was measured at different stages of growth. Activity of OxdC in *L. plantarum* pLp_0373sOxdC was 0.01 U/mg proteins, 0.02 U/mg proteins, and 0.05 U/mg proteins for early exponential, late exponential and late logarithmic phases, respectively. *L. plantarum* pLp_3050sOxdC showed 0.002 U/mg proteins, 0.01 U/mg proteins, and 0.02 U/mg proteins during three different stages. It also indicates that enzymatic activity of OxdC in supernatant of recombinant *L. plantarum* WCFS1pLp_0373OxdC and WCFS1pLp_3050OxdC was increased directly proportional to the growth of the strains (Figure 3). The higher activity of OxdC from recombinant *L. plantarum* WCFS1 harboring the plasmid pLp_0373OxdC indicates that secretion of OxdC protein is higher than the strain harboring the plasmid pLp_3050sOxdC. No activity was determined in the supernatant of *L. plantarum* pSip-OxdC.

The OxdC activity in cellular fraction of recombinant strains (OD_600_ ~ 1.7) showed 2.8 U/mg proteins, 0.18 U/mg proteins, and 0.21 U/mg proteins for pSip-OxdC, pLp_0373sOxdC, and pLp_3050sOxdC, respectively. However, no activity was observed in both supernatant and cellular fraction of wild type *L. plantarum* WCFS1. To assess the secretion efficiency, intracellular OxdC activity was measured at OD_600_ ~ 1.7. Secretion efficiencies are expressed as the percentage of the total OxdC activity present in the cell-free supernatants at OD_600_ ~ 1.7. The SP Lp_0373 showed highest secretion efficiency into the medium with 22%, whereas SP Lp_3050 showed only 9% which suggest that ~78% and ~91% of intracellular OxdC were the combination of processed and unprocessed forms of OxdC in recombinant *L. plantarum* pLp_0373sOxdC and pLp_3050sOxdC, respectively (Table 3). 

### 3.3. Purification and Biological Activity of Recombinant OxdC

**Figure 4:**
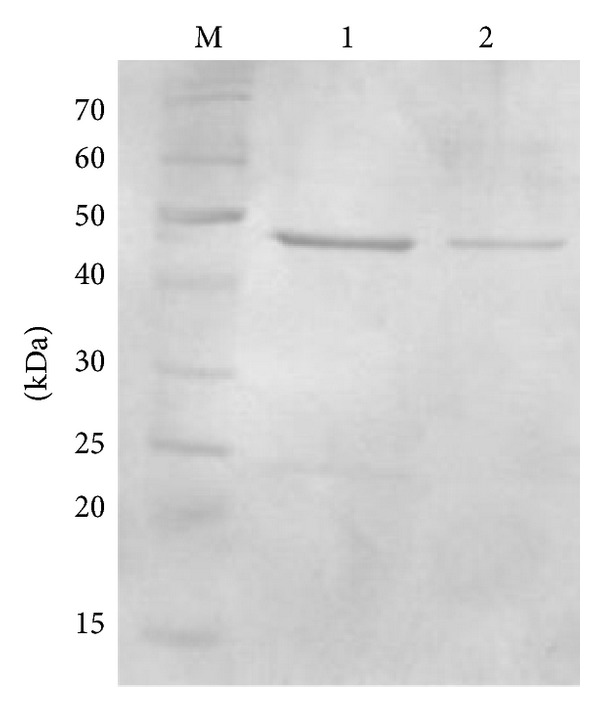
SDS-PAGE showing the purified OxdC protein from culture supernatant of recombinant strains by FPLC. lane M, molecular weight marker in KDa; lane 1, purified OxdC from strain harboring plasmid pLp_0373OxdC; lane 2, purified OxdC from strain harboring plasmid pLp_3050OxdC.

**Figure 5:**
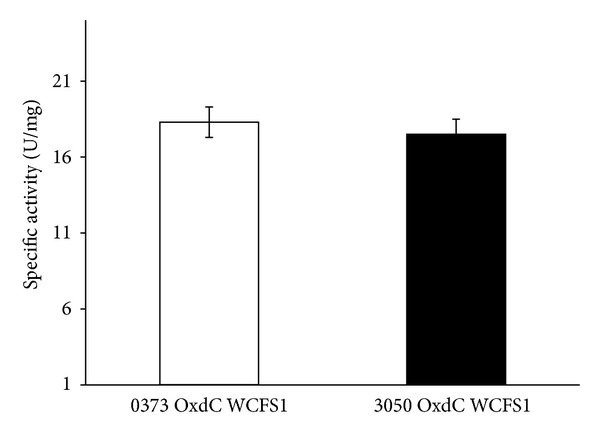
Specific activity of purified recombinant oxalate decarboxylase from supernatant of recombinant *L. plantarum* WCFS1 (0373 OxdC WCFS1 and 3050 OxdC WCFS1 indicate the OxdC purified from strain harboring the plasmid pLp_0373sOxdC and pLp_3050sOxdC, respectively), both the results are the mean of three independent experiments; the error bars indicate the standard deviation. Enzyme activities are expressed in Units/mg of protein.

Purified OxdC by FPLC from the supernatant of recombinant* L. plantarum* WCFS1 was resolved in SDS-PAGE (Figure 4). As expected the purified protein band shows calculated molecular weight corresponding to 44 KDa in SDS-PAGE, which indicates the correct cleavage of signal peptides by signal peptidase-I. OxdC secreted into the medium by pLp_0373sOxdC was found to be higher, which has approximately the concentration of 8–12 **µ**g/mL and the specific activity of the purified OxdC from the supernatant of *L. plantarum* pLp_0373sOxdC was 18.3 U/mg protein. The specific activity of secreted protein which obtained from *L. plantarum* pLp_3050sOxdC was 17.5 U/mg protein (Figure 5) and concentration of OxdC protein in supernatant was 5–8 **µ**g/mL.

### 3.4. Oxalate Degradation by Recombinant *L. plantarum* Strains Expressing Secretary OxdC

**Figure 6:**
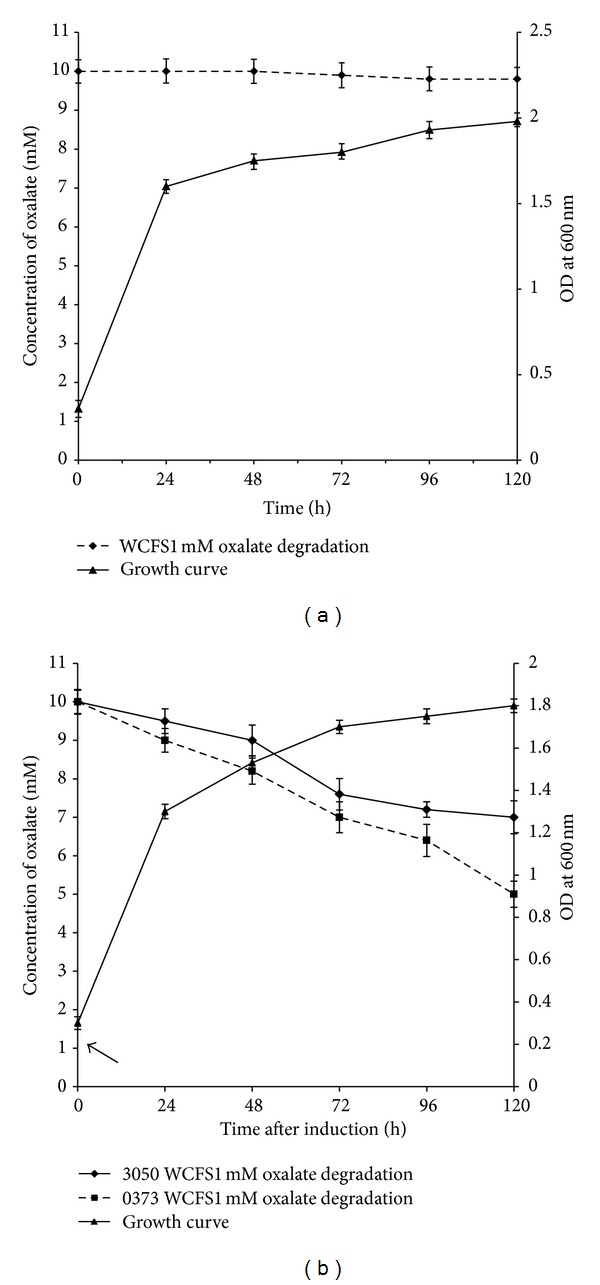
Oxalate degradation capabilities of wild type and recombinant *L. plantarum* WCFS1. (a) Oxalate degradation ability of wild type *L. plantarum* WCFS1 corresponding simples of WCFS1 mM oxalate degradation and growth curve indicate concentration of oxalate reduction in supernatant against the growth of the wild type *L. plantarum* WCFS1 strain, respectively; (b) oxalate degradation capability of recombinant strains corresponding simples of 3050 WCFS1, 0373 WCFS1 mM oxalate degradation and growth curve indicate concentration of oxalate reduction in supernatant of recombinant *L. plantarum* WCFS1 strains harboring plasmid pLp_3050OxdC and pLp_0373OxdC, respectively, against the growth of the strains. Arrow indicates the point of induction using peptide (sppIP) at OD_600_ ~ 0.3.

The oxalate degradation ability of *L. plantarum *WCFS1 carrying plasmids pLp_0373sOxdC, pLp_3050sOxdC was evaluated in the presence of 10 mM potassium oxalate. The results showed reduction in oxalate level in the medium of recombinant *L. plantarum,* while the medium of wild type *L. plantarum* showed no reduction in oxalate level. The oxalate level in the medium of *L. plantarum* WCFS1 harboring the plasmid pLp_0373sOxdC and pLp_3050sOxdC decreased from 10 mM to around 5 mM (50%) and 7 mM (30%), respectively, in 120 h incubation. The result indicates that the oxalate present in the medium of recombinant strain was degraded as a consequence of secreted OxdC. Results also indicate that the oxalate degrading ability increased proportionately with the growth of the strains (Figures 6(a) and 6(b)).

## 4. Discussion

The present study aims to develop a recombinant LAB that can be used as a possible tool for the degradation of intestinal oxalate and prevention of hyperoxaluria and calcium oxalate stone disease in human. Here, we developed a genetically modified *L. plantarum* bacterial system for the delivery of heterologous OxdC at biologically active condition for therapeutic use. *L. plantarum* is found in many habitats, including dairy and meat products and plant and vegetable fermentations and in the gastrointestinal tract and oral cavity of humans [35]. *L. plantarum* WCFS1 is a single colony isolate of *L. plantarum* NCIMB8826, which exhibits efficient secretion of heterologous protein compared with other *L. plantarum* strains. Kleerebezem et al. [25] identified 93 proteins with putative signal peptidase-I cleavage site in the genome of *L. plantarum* WCFS1. The signal peptide functionality of 76 sec-type signal peptides from *L. plantarum* WCFS1 that were predicted to be cleaved by signal peptides-I was studied by Mathiesen et al. [24]. They also reported that 82% of 76 tested SPs led to secretion of NucA. In addition the SPs Lp_3050 and Lp_0373 of this bacterium showed higher secretion of heterologous protein [31].

In our study, activity of OxdC in culture supernatant of *L. plantarum* pLp_0373sOxdC was higher than the recombinant strain of pLp_3050sOxdC, which is similar to the results on amylase activity reported by Mathiesen et al. [31]. Secretion efficiency of OxdC in the strain containing pLp_0373sOxdC was 70% higher than pLp_3050sOxdC. This higher secretion indicates that this signal peptide is most promising for future use. However, the overall picture emerging from previous studies showed the difficulties on rationalizing the secretion levels and secretion efficiencies [36–38]. Thus, it is clear that secretion levels and secretion efficiency are not only steered by the SP but also by the secretion target. The present work enables the simple downstream purification step of OxdC, which explores the possibility of large scale production of the OxdC protein for industrial and biotechnological applications.

The most extensively investigated form of OxdC is that isolated from *B. subtilis*. The expression of this enzyme in the cell wall of *B. subtilis* is found to be profound during acidic stress conditions. This bacterial enzyme has been the model for numerous studies aimed at deciphering the catalytic mechanism of oxalate decarboxylase. Recent studies have focused on the use of OxdC as a potential mechanism to decompose intestinal oxalate in humans. After the discovery that the *YrvK* gene of *B. subtilis* encodes the 44 kDa oxalate decarboxylase [34], investigators were able to develop recombinant *Escherichia coli* (*E. coli*) expressing this gene. Potent oxalate degrading activity was observed when the recombinant *E. coli *was administered orally to hyperoxaluric rat models [20]. Extrapolating from these findings, Grujic et al. [21] demonstrated that oral administration of recombinant *B. subtilis* OxdC to hyperoxaluric mice resulted in decreased oxalate urinary excretion and prevention of nephrocalcinosis. Therefore, we undertook this study to degrade the intestinal oxalate by secreting OxdC by probiotic strain *L. plantarum.*

The reductions of 30% and 50% of oxalate concentration were observed in the supernatant of recombinant strains during the 120 h incubation period but there was no change in oxalate concentration in wild type *L. plantarum*. The final pH of the supernatant of recombinant strain of *L. plantarum* has reached nearly to pH 4.0 (date not shown). Tanner and Bornemann [34] reported the enzyme OxdC was induced in acidic growth media and shows maximum activity at pH 5.0, and also found 70% activity at pH 3.0. Turroni et al. [39] experiment of pH controlled batch fermentations revealed that acidic conditions were a prerequisite for a significant oxalate degradation rate. Although, the pSIP vectors have been successfully applied for the production of different proteins in *L. plantarum *[23, 30, 40, 41], the heterologous OxdC quantity in *L. plantarum* may be further increased by usage of synthetic propeptide residues for the high oxalate degradation. The fusion of a short synthetic propeptide between the SP and the mature moiety is another innovative biotechnological tool to enhance protein secretion. One such synthetic propeptide LEISSTCDA, a nine amino acid peptide, which was shown to enhance the secretion efficiency of several heterologous proteins in *Lactococcus lactis*: NucB, NucT [42]. Even though, secretion efficiency is fairly normal, the another possibility to increase the secretion efficiency is to test other SPs from the library made previously and find the optimal SPs that lead to higher secretion efficiency. It is difficult to compare the present observation with the results of previews studies, since the studies published so far on engineered secretion in lactic acid bacteria differ with respect to the genetic constructs and host strains used [42].

The accumulation of intracellular precursors and the high total level of OxdC activity may be due to the fact that intracellular protein is better protected from proteolytic degradation than secreted OxdC protein [24]. While the purified OxdC from culture supernatant of recombinants *L. plantarum* harboring the plasmid pLp_0373sOxdC and pLp_3050sOxdC shows the specific activity of 18.3 U and 17.5 U/mg protein, respectively, it is almost similar to the activity of OxdC purified from the recombinant *E. coli* BL21 [26]. Mufarrij et al. [43] reported that commercially produced form of oxalate decarboxylase (Oxazyme) degrades the oxalate completely in the intestinal buffer (pH 6.5) and oxalate derived from potassium oxalate in the gastric buffer (pH 3.6) was profoundly digested by Oxazyme. Adding Oxazyme also substantially reduced the oxalate content of both whole and homogenized spinach preparations, in either buffer. These *in vitro* findings demonstrate that Oxazyme can metabolize oxalate in both simulated gastric and small intestinal environments. Cowley et al. [22] evaluated the toxicity studies in rats and dogs by orally administered *B. subtilis* OxdC enzyme. The results of the study showed no observed adverse effect in both of the animals administered OxdC (720.8 mg/kg/d) in rats and (187.2 mg/kg/d) in dogs.

Overexpressing OxdC in *L. plantarum* would enable the recombinant strain to efficiently degrade the oxalate in the gut environment. However, certain optimization needs to be done for its application *in vivo*. The optimization may include replacing the antibiotic resistance marker with a food-grade selection marker that will allow to study directly on animal models. This was recently demonstrated with the pSIP vectors using *alr *gene as plasmid selection marker instead of the erythromycin that has been applied for overexpression of *β*-galactosidase [44]. Similar kind of studies needs to be performed in future to harness real benefits of genetically modified *L. plantarum* strains for humans.

## 5. Conclusions

In conclusion, the results indicate that oxalate present in the extracellular environment could be degraded by the recombinant strains secreting heterologous oxalate decarboxylase. Hence, the recombinant *Lactobacillus *bacteria developed in this study with further modification like change of constitutive promoter, usage of synthetic propeptide sequence, biological containment status, and improved gastrointestinal (GI) track survival will enable us to degrade the oxalate available in intestine of human for therapeutic use. Based on the genetics tools availability the manipulation of recombinant *L. plantarum* WCFS1 with the biologically contained status will ease in future focusing.

## Figures and Tables

**Table 1:** Bacterial strains and plasmids used in this study.

Strains and plasmids	Characteristics^a^	Source/references
Strains		
* E. coli* DH10B	Host strain for cloning commercial	Invitrogen
* L. plantarum *WCFS1*	Host strain, plasmid-free, silage isolate	Kleerebezem et al. (2003) [25]
Plasmid		
pLp_0373sAmyA	p256/pUC(pGEM)ori; P_sppA_; Lp_0373; *amyA*; Em^r^	Mathiesen et al. (2009) [24]
pLp_3050sAmyA	p256/pUC(pGEM)ori; P_sppA_; Lp_3050; *amyA*; Em^r^	Mathiesen et al. (2009) [24]
pLB36	pET 32a *oxdC;* Am^r^	Just et al. (2004) [26]
pSip-OxdC	p256/pUC(pGEM)ori; P_sppA_; *oxdC*; Em^r^	Kolandaswamy et al. (2009) [23]
pLp_0373sOxdC	p256/pUC(pGEM)ori; P_sppA_; sp_Lp_0373 _fused to the* oxdC*; Em^r^	This work
pLp_3050sOxdC	p256/pUC(pGEM)ori; P_sppA_; sp_Lp_3050_fused to the *oxdC*; Em^r^	This work

^a^For strains, genotypic and phenotypic characteristics are given; for plasmid, plasmid and cloned-cassette characteristics are given; Em^r^, Am^r^: resistance to erythromycin and ampicillin, respectively.

*L. plantarum WCFS1 is a single colony isolate of strain NCIM8826 (Kleerebezem et al., 2003 [25]).

**Table 2:** Primers and induction peptide used in this study.

Primers/peptide	Sequences (5′→3′)^a^
OXDC sense	5′-GAGAGTCGACATGAAAAAACAAAATGACATTCCGC-3′ (*Sal *I)
OXDC antisense	5′-GGAATTCGTGGTGGTGGTGGTGGTGTTATTTACTGCATTTCTTTTTCACTA-3′ (*EcoR *I)
PsppA sense	5′-GTCACTAACCTGCCCCGTTA-3′
SppIP	MAGNSSNFIHKIKQIFTHR*

^a^The restriction sites are underlined;*aminoacid sequences of induction peptide (SppIP).

**Table 3:** Oxalate decarboxylase (OxdC) activity in concentrated culture supernatant and cell lysate of various recombinant *L. plantarum* WCFS1 strains^a^.

Name of strain (plasmid)	Protein localization	OxdC (U/mg protein) activity in culture supernatant at OD_600_ = 1.7	OxdC (U/mg protein) activity in cell fraction at OD_600 _= 1.7	Secretion efficiency* (%)
WCFS1 (Control)	NP	NA	NA	ND
WCFS1 (pSIP-OxdC)	Intracellular	NA	2.8 ± 0.2	ND
WCFS1 (pLp_0373sOxdC)	Extracellular	0.05 ± 0.01	0.18 ± 0.07	22
WCFS1 (pLp_3050sOxdC)	Extracellular	0.02 ± 0.01	0.21 ± 0.07	9

^a^Data are mean of three independent experiments, NP: no production, NA: no activity, and ND: not determined, and * secretion efficiency were determined at OD_600_ = 1.7 between the culture supernatant and cell fraction of respective strains.
